# Reverse pH-dependent fluorescence protein visualizes pattern of interfacial proton dynamics during hydrogen evolution reaction

**DOI:** 10.1038/s41598-023-44758-4

**Published:** 2023-10-15

**Authors:** Trisha Diba Farha, Samyoung Kim, Mieko Imayasu, Atsushi Miyawaki, Hidekazu Tsutsui

**Affiliations:** 1 School of Materials Science, Japan Advanced Institute of Science and Technology, Nomi, Ishikawa 923-1292 Japan; 2 Biotechnological Optics Research Team, RIKEN Center for Advanced Photonics, Wako, Saitama 351-0198 Japan

**Keywords:** Proteins, Biophysical chemistry, Proteins

## Abstract

Reverse pH-dependent fluorescent protein, including dKeima, is a type of fluorescent protein in which the chromophore protonation state depends inversely on external pH. The dependence is maintained even when immobilized at the metal-solution interface. But, interestingly, its responses to the hydrogen evolution reaction (HER) at the interface are not reversed: HER rises the pH of the solution around the cathode, but, highly active HER induces chromophore deprotonation regardless of the reverse pH dependence, reflecting an interface-specific deprotonation effect by HER. Here, we exploit this phenomenon to perform scanning-less, real-time visualization of interfacial proton dynamics during HER at a wide field of view. By using dKeima, the HER-driven deprotonation effect was well discriminated from the solution pH effect. In the electrodes of composite structures with a catalyst, dKeima visualized keen dependence of the proton depletion pattern on the electrode configuration. In addition, propagations of optical signals were observed, which seemingly reflect long-range proton hopping confined to the metal-solution interface. Thus, reverse pH-dependent fluorescent proteins provide a unique tool for spatiotemporal analysis of interfacial proton dynamics, which is expected to contribute to a better understanding of the HER process and ultimately to the safe and efficient production of molecular hydrogen.

## Introduction

Reverse (or inverse) pH-dependent fluorescent protein is a type of fluorescent protein in which the protonation state of the hydroxyl group of the chromophore depends unconventionally on pH, with the protonated state (–OH) predominating at high pH and the deprotonated state (–O^−^) at low pH. This is in contrast to *Aequorea victoria* green fluorescent protein (GFP) and many GFP-like proteins, where the protonation state straightforwardly follows the external pH, with the protonated and deprotonated states predominating at low and high pH, respectively^1,2^. Examples of the reverse pH-dependent fluorescent protein include ratiometric pHluorin^3^, iR pHluorin^4^, and Keima^5^. It is generally considered that the protonation states of the chromophore surrounding residues affect the complex hydrogen bonding network of the protein cavity, resulting in a reverse pH dependence^6^. Based on those proteins, various techniques have been developed to quantitatively analyze dynamic intracellular events such as secretion, synaptic transmission, and autophagy^3,4^.

The reverse dependency on pH is maintained even when the proteins are immobilized at the interface between solution and metal. But, interestingly, their responses to the hydrogen evolution reaction (HER) at the interface are not reversed^7,8^. Here, HER is one of the half-reactions of electrochemical water splitting. HER raises the pH of the solution around the metal which works as a cathode, but regardless of whether its pH dependence is normal or reversed, highly active HER promotes deprotonation of the chromophore^8^. It is thought that an interface-specific proton pathway is formed between the fluorescent protein and the metal surface, and the protonation state of the chromophore of the fluorescent protein is directly affected by the interfacial proton reduction^8^. In this study, we took such a unique aspect of reverse pH-dependent fluorescence protein to perform scanning-less, real-time visualization of interfacial proton dynamics during HER at a wide field of view.

To highlight the significance of the reverse pH dependence, we used the fluorescence proteins, Venus and dKeima. Venus is a variant of yellow fluorescence protein and exhibits conventional pH dependency^9^. The deprotonated chromophore emits bright fluorescence at 528 nm upon blue light excitation whereas the protonated state is non-fluorescent. dKeima is a coral-derived red fluorescence protein and exhibits reverse pH dependency^5^. The protonated and deprotonated states have excitation peaks at 440 nm and 586 nm, respectively, and both emit at 620 nm. The pKa values for dKeima and Venus immobilized at the interface were measured as 6.2 and 6.3 and were close to 6.5 and 6.0, which were the values at the solution from the original reports^5,9^. The basic properties of these proteins are summarized in Fig. S1.

**Figure 1:**
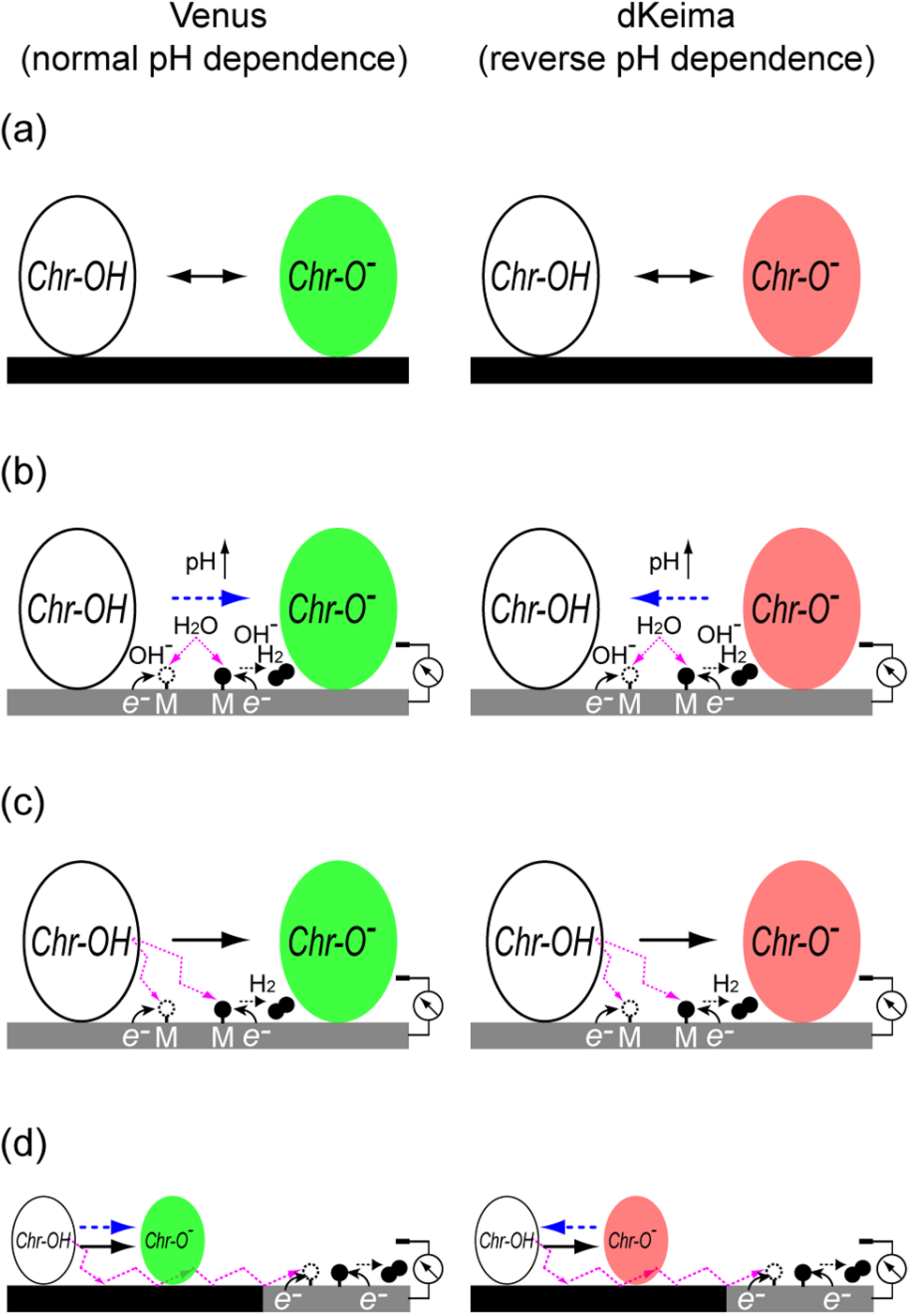
Mechanisms of fluorescence modulations in Venus and dKeima by HER. (**a**) Chromophore protonation equilibrium at the resting state: Chr–OH ↔ Chr–O^−^ + H^+^. For both Venus and dKeima, fluorescence from the deprotonate state (Chr–O^−^) was detected in the present experimental condition. (**b**) Alkalization due to Volmer (H_2_O + M + e^−^ → M–H + OH^−^) and Heyrovsky (H_2_O + M–H + e^-^ → M + H_2_ + OH^−^) steps exert the solution pH effect, inducing deprotonation in Venus and protonation in dKeima. (**c**) Donation of proton through the interface-specific proton path, by which the chromophore deprotonation is facilitated both in Venus and dKeima. (**d**) Proton donation via the long-distance proton hopping to the HER active site. M: metal, white circle: proton binding site; black filled circle: hydrogen.

Figure 1 illustrates the principle of imaging. At the resting condition of null bias voltage, the chromophore’s phenolic hydroxyl group exists at the original equilibrium between the protonated (ChrOH) and deprotonates (ChrO^−^) states (Fig. 1a). Activation of HER shifts the protonation equilibrium by two different effects. First, upon HER activation, both Volmer and Heyrovsky steps induce alkalization and the eventual equilibrium shift toward deprotonation in Venus whereas protonation occurs in dKeima (Fig. 1b). We define this process as the solution pH effect. Secondly, as HER is enhanced, the fluorescence protein itself acts as a proton donor through the interface-specific proton path, by which the chromophore deprotonation is facilitated regardless of the type of pH-dependency (Fig. 1c). We define this process as the interface-specific deprotonation effect. Furthermore, the chromophore deprotonation is facilitated by HER at a distance from the protein, which is suggested to be via long-distance proton hopping confined at the interface, as will be documented later (Fig. 1d).

## Results

### Fluorescence protein as an indicator of proton dynamics during HER

**Figure 2:**
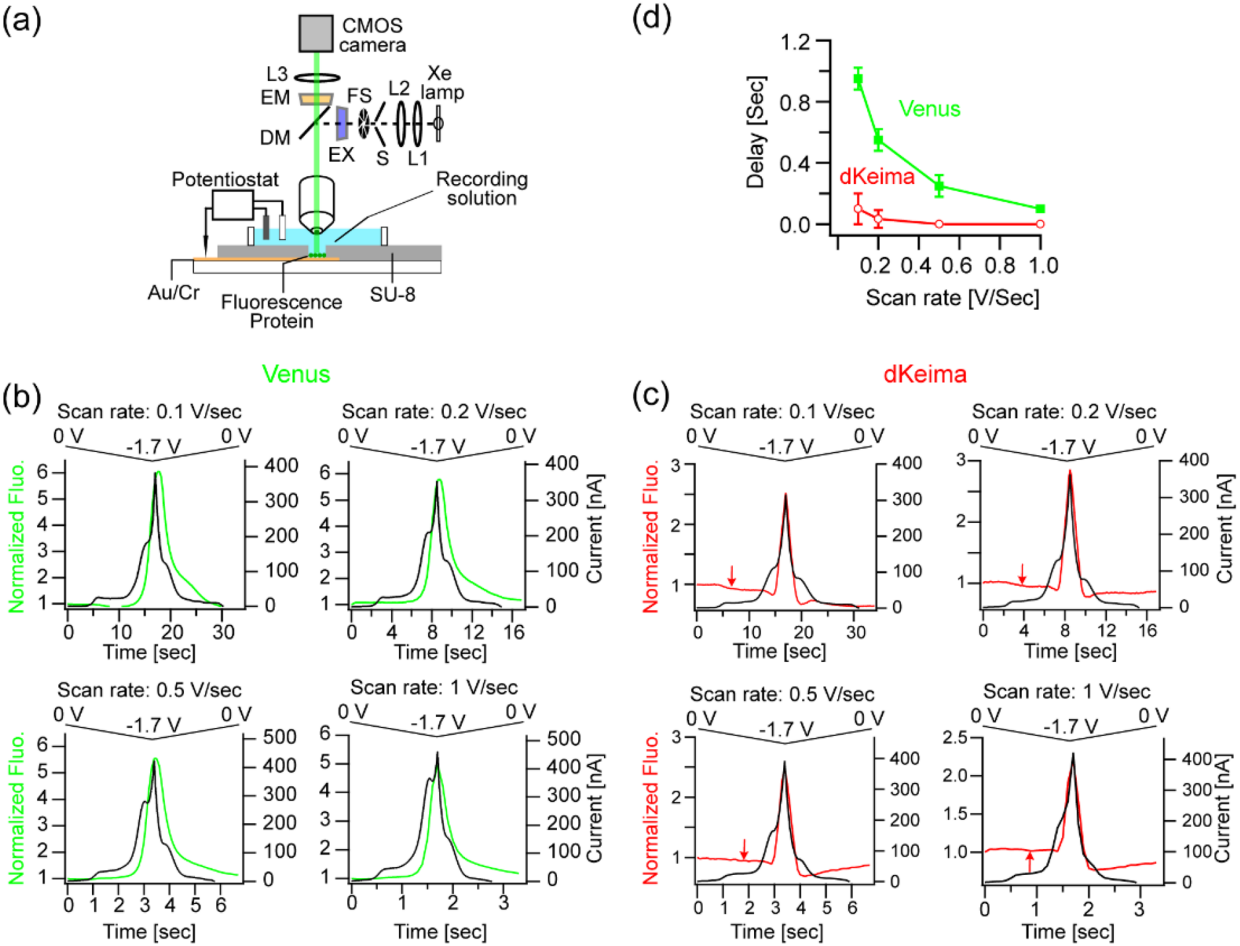
Correlations between HER and fluorescence protein emission at the four different scan rates. (**a**) The configuration of the experiments. (**b**) Current and fluorescence responses in the electrode immobilized with Venus. (**c**) The responses in the electrode with dKeima. (**d**) Delay of the optical signal to the electrode current plotted versus the scan rate.

To evaluate the performance of these proteins as an indicator of interfacial proton dynamics, we fabricated gold microelectrodes with a diameter of 50 μm, immobilized fluorescence proteins, and then performed simultaneous electrochemistry and fluorescence microscopy (Fig. 2a). The recording solution consisted of 20 mM HEPES (pH 7.4; adjusted with NaOH) and 1 mM NaCl. First, the correlation between HER and the fluorescence responses was examined by scanning the potential from 0 to − 1.7 [V vs. Ag/AgCl] at the four different scan rates: 0.1, 0.2, 0.5, and 1 [V/s]. Blue (489–505 nm) and green light (510–560 nm) were used for the excitation of deprotonated forms of Venus and dKeima, respectively; thus fluorescence increase indicates deprotonation in both of the proteins. As an overall trend, HER and the optical signals were well correlated in all the scan rates in Venus (Fig. 2b) and dKeima (Fig. 2c). Here, a current of 100 nA corresponds to a current density of ~ 5.1 mA/cm^2^ when averaged over the electrode area. Notably, the directions of the major optical signals at the large negative voltage (− 1.5 to − 1.7 V) in these two proteins were identical (i.e. fluorescence increase) despite their opposite pH-dependences, indicating that the major signal is contributed by the HER-driven interfacial deprotonation effect rather than the solution pH effect. Full widths at half maximum (FWHM) of Venus signal for the scan rate of 0.1, 0.2, 0.5, and 1 [V/s] were: 3.3 ± 0.3 (average ± s.d; n = 4), 1.7 ± 0.1(n = 2), 0.76 ± 0.05 (n = 2), and 0.38 ± 0.01 (n = 2) [s], respectively. FWHMs of dKeima signals were: 1.8 ± 0.1 (n = 4), 1.0 ± 0.1(n = 3), 0.43 ± 0.05 (n = 3), and 0.23 ± 0.04 (n = 3) [s], respectively. Thus, dKeima produced sharper responses than Venus (Welch’s t-test, *p* < 0.05). This is because the interfacial deprotonation effect and the solution pH effect work constructively in the Venus signal but destructively in dKeima. At low negative voltage (> − 1.4 V), a slight fluorescence decrease was evident in dKeima (Fig. 2c, arrows), reflecting the dominance of the solution pH effect in those voltage ranges. Figure 2d shows the delay of optical signals versus the scan rate as measured by the cross-correlation analysis. The sharper responses of dKeima resulted in a shorter delay when compared to Venus. These high correlations between HER and optical signals validated that fluorescent protein emission can be used as an indicator of interfacial proton dynamics during HER.

### Proton depletion pattern in a heterogeneous reaction environment

**Figure 3:**
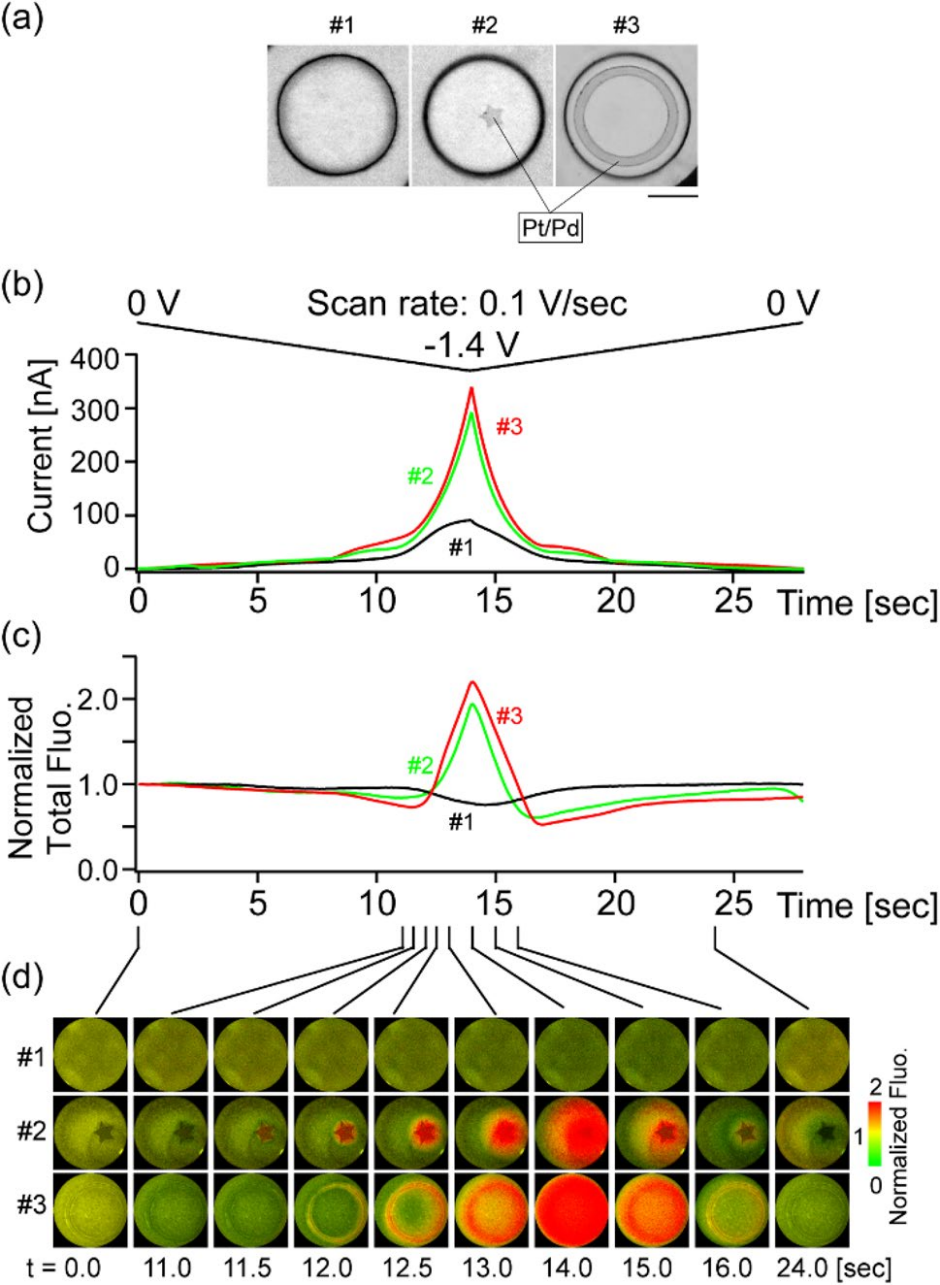
Spatiotemporal dynamics of dKeima signals in the gold electrodes with or without the Pt/Pd subregion revealed by dKeima imaging, (**a**) Examples of the electrodes analyzed. Bar = 20 μm. (**b**) A plot of the current responses versus time during the voltage scan to − 1.4 V. (**c**) The total fluorescence versus time. (**d**) Pseudo-colored presentations of the normalized dKeima responses.

We then sought to visualize proton dynamics in heterogeneous environments. To this end, a star- or ring-shaped island of platinum/palladium (Pt/Pd) was fabricated onto the gold surface (Fig. 3a). We used dKeima with reverse pH dependence as an indicator since it allows straightforward discrimination between the interfacial deprotonation effect and the solution pH effect. Figure 3b,c show the representative responses of the currents and the total fluorescence, respectively, elicited by the potential scan from 0 to − 1.4 V. The current was less than 100 nA in the bare gold electrode (Fig. 3b, #1), and was enhanced more than three-fold in the electrodes with Pt/Pd islands, validating their highly catalytic nature (Fig. 3b, #2, 3). Total dKeima fluorescence in the bare gold electrode decreased slightly (Fig. 3c, #1), showing limited HER and the occurrence of the solution pH effect only. This observation was also consistent with the result in Fig. 2 around − 1.4 V. By contrast, in the electrodes with Pt/Pd islands, a robust increase of total fluorescence was observed (Fig. 3c, #2, #3), reflecting the HER-driven interfacial deprotonation effect. Importantly, while the current and total fluorescence profiles were similar in these electrodes (Fig. 3c, #2, #3), dKeima imaging visualized the difference in the patterns of interfacial proton dynamics (Fig. 3d; Movies S1–S3). Initially, a decrease in fluorescence was detected selectively from the region near the Pt/Pd catalyst (Fig. 3d #2, #3; t = 11.0 s), which was then followed by a fluorescence increase. dKeima imaging thus visualized the solution pH effect and the eventual rise in the HER-driven interfacial deprotonation effect.

**Figure 4:**
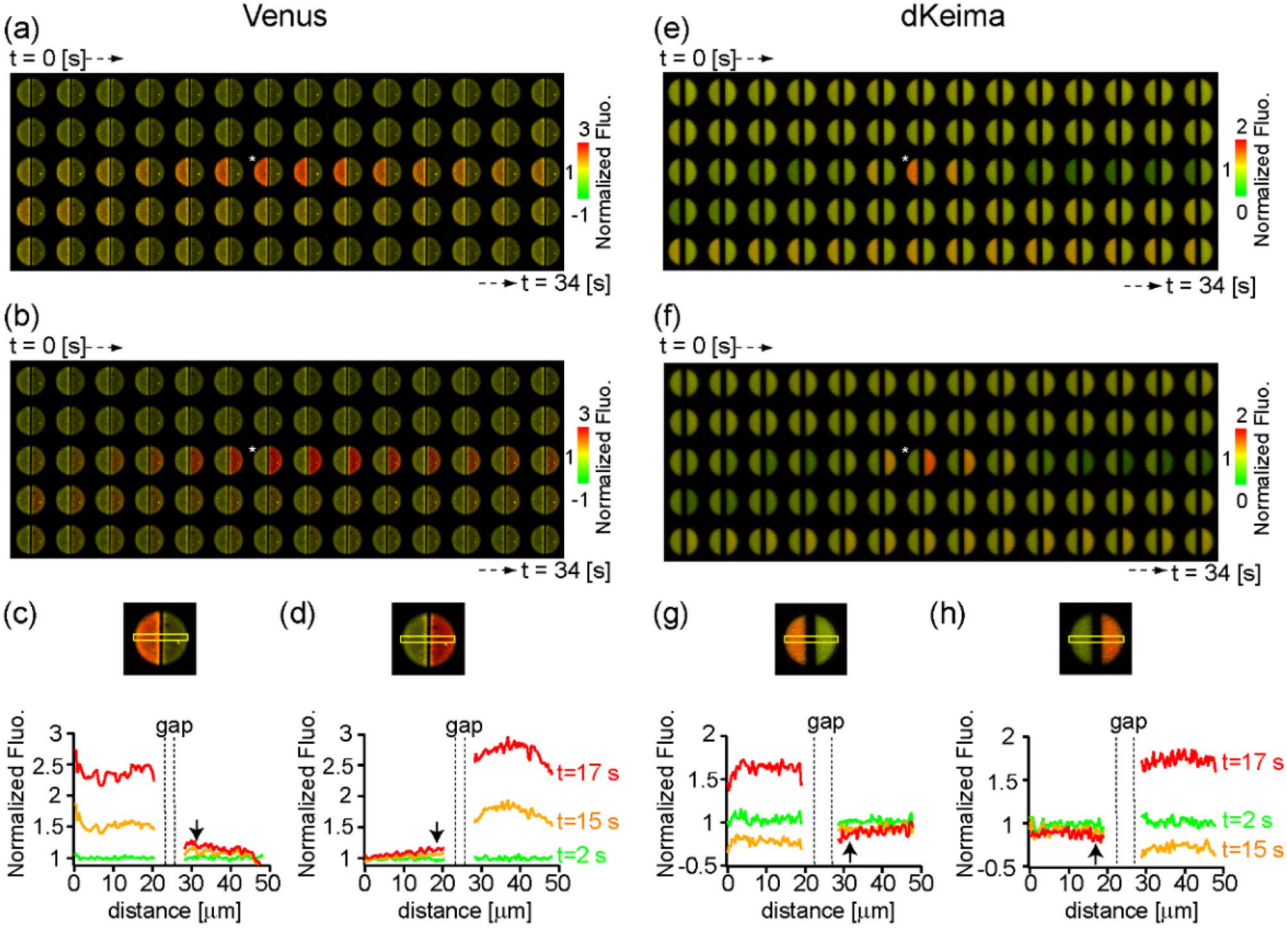
The spatial correlation between HER and fluorescence protein emission. (**a**, **b**) Representative response of Venus fluorescence elicited by the potential scan at the left (**a**) or right (**b**) electrode. The asterisks indicate the image frames at the negative peak potential (− 1.7 V). (**c**, **d**) Profiles of the fluorescence response along the horizontal axis in the region indicated by the yellow line. The left electrode is active in (**c**) and the right in (**d**). (**e**–**f**) The same set of data for dKeima.

Furthermore, dKeima imaging highlighted propagation of the optical signal from the proximal to distal to the Pt/Pd is-lands (Movies S2, S3). To ask whether the bulk solution could mediate the propagation, we fabricated a coffee-bean-shaped split microelectrode—a pair of electrically independent semi-circular electrodes with a 5–8 μm wide gap. After protein immobilization onto both of the electrodes, fluorescence imaging was performed while scanning the bias voltage at one of the electrodes to activate HER and leaving the other open-circuited. Here, we refer to these as the active and the resting electrode, respectively. When Venus was used as an indicator, a robust increase of fluorescence was confirmed at the active electrode. The propagation of the optical signal to the resting electrode was not detected (Fig. 4a,b; Movies S4, S5). In a careful analysis of the profiles, we found a slight increase of fluorescence in the resting electrode at the region near the gap (Fig. 4c,d; arrows). In the experiment using dKeima, on the other hand, fluorescence at the active electrode decreased at a low negative voltage (> − 1.4 V) and then robustly increased at a large negative voltage (< − 1.4 V), which reproduced the detection of the solution pH effect and eventual activation of the HER-driven interfacial deprotonation effect (Fig. 4e,f; Movies S6, S7). In the resting electrode, a slight decrease in intensity was observed upon the application of a large negative voltage (< − 1.4 V) at the active electrode (Fig. 4g,h), which thus signified the solution pH effect. Taken together, the measurements using the split electrodes suggested that the solution pH effect tends to surmount the electrode gap whereas the HER-driven proton depletion does not. So we hypothesized as follows; the propagation of HER-driven proton depletion originating from the Pt/Pd islands (Fig. 3d) is mediated by the Grotthuss-like proton transfer confined at the interface. Based on this assumption, we performed a quantitative analysis of the time-series image data in the rotationally symmetric type #3 electrodes (Fig. S2). We focused on the time point, t = 12.5 s, and the velocities at which the ΔF/F = 10% contour lines propagate were measured for the rectangular areas of 5 μm wide, vertically and horizontally from the center. The propagation rates were measured as 14 ~ 26 μm/s. Comparing the current profile at electrode #3 with that at #1, i.e. no Pt/Pd catalyst, (Fig. 3c), we assumed a simplified situation that HER only occurs at the Pt/Pd catalyst region. It was also assumed that the propagation of dKeima signals in the narrow region of interests of 5um width can be approximated by a one-dimensional diffusion equation. Under such simplification, the diffusion coefficient was estimated as the ratio of the time derivative of dKeima fluorescence (ΔF/ΔT) and the second-order derivative with respect to the direction of propagation (Δ^2^F/(Δx)^2^). The diffusion coefficients measured for the four directions at t = 12.2 and 12.3 s ranged 16–180 μm^2^/s (Fig. S2).

## Discussion

The present study demonstrated that the reverse pH-dependent fluorescence protein permits spatiotemporal analysis of interfacial proton dynamics during HER. While the solution pH during HER has been deeply focused through the various approaches^10–12^, the effect at the interface and that at the solution has not been explicitly distinguished. We used this reverse pH dependence to distinguish the HER-driven interfacial deprotonation effect from the solution pH effect. The present observation that the interfacial deprotonation effect did not cross the physical gap at the split electrode is consistent with the previous observation that the interface signal was not affected by the intense flow of the solution^8^. Proton behaviors at the interface have been thought to play key roles in various reactions^13–15^, so the measurements will provide critical information to understand the reaction environment. Since small organic fluorophores generally do not exhibit such reverse pH dependence, the present approach may only be achieved with those proteins. Although the protein immobilized could affect the reaction environment of interest as in many other assays where probes are introduced externally, it may be also possible to control the amount of protein by modifying the immobilization protocol and to study its effect on the reaction environment^7^. Comparison with the existing scanning electrochemistry techniques^16–18^ highlights various specific features. The most basic point is that the fluorescence protein signal reflects the behaviors of the donors of the reaction species (i.e. proton) at the interface whereas the scanning probe detects currents reflecting actual reduction/oxidation reactions. So these two-dimensional measurements in principle are complementary to each other. Also, the present approach enables a scanning-less visualization of a single event, which can be significant when the trial-by-trial differences in the reaction environment can be critical. Concerning spatial resolution, scanning electrochemistry allows very high-resolution mapping at the submicron resolution, but it may not be easy with fluorescence microscopy to exceed the diffraction limit. The capability of quantitative analysis should also be critical. In theory, protein densities and depletion rates could be quantified with ratiometric imaging of dKeima using a dual excitation light source as its protonated and deprotonated states have specific excitation peaks at 440 nm and 586 nm, respectively. Accurate knowledge of the molar extinction coefficient and fluorescence quantum yield of proteins as well as the kinetics of the protonation-deprotonation at the interface should be also essential for such purpose. An explicit characterization of the proton dynamics of the whole system consisting of the interface and the solution would be significant to understanding the reaction environment in further depth. To this end, for example, three-dimensional imaging of the reaction environment in which dKeima indicator is present both at the interface and in the solution may give important insights. More ultimately, building a model for proton dynamics taking into account diffusion rates and interfacial pH gradients would provide deep quantitative analysis. So it would be worthwhile to combine the modeling approach^12^ for such a future direction. The present approach is most compatible with neutral to weak alkaline conditions. Achieving efficient HER at near-neutral conditions is significant in realizing safe and low-cost production of molecular hydrogen^19–21^, one of today’s pressing issues^22,23^. Therefore, reverse pH-dependent fluorescence proteins placed at the interface would provide a unique platform for analyzing and understanding interfacial proton dynamics in such reaction environments.

## Methods

### Electrode fabrication

Borosilicate coverslips (22 × 32 mm) intensely cleaned with acetone were sputtered with Cr and Au to the thickness of 30 nm and 200 nm, respectively. The ion beam sputtering system (EIS-220, Elionix, Tokyo, Japan) was used for sputtering. The typical sputtering time was 20 min for each process. Photolithography was then performed to form the electrode lines and connection pads by using the photoresist S1818 (MicroChem, MA, USA). After cleaning with piranha solution, to form the patterned windows for Pt/Pd islands, the substrate was subject to secondary lithography with the photoresist SU8-3005 (MicroChem, MA, USA). Pt/Pd was then sputtered to ~ 100 nm using the ion sputter (E-1030, Hitachi, Japan). The sputtering time was typically 180 s. To peel off the SU8 layer of the windows, the substrate was treated with N-Methyl-2-pyrrolidone (TCI, Tokyo, Japan) at 72 ºC for several hours After cleaning with piranha solution, the third lithography was done to form the insulator layer as well as the circular electrode openings with a diameter of 50 μm. A silicone washer with an inner diameter of 6 mm and a height of 1 mm was glued with poly-dimethylsiloxane to form the recording chamber. The whole device was kept in a desiccator and treated with plasma cleaning before use (PDC-32G, Harrick Plasma, N.Y., USA).

### Protein preparation and immobilization

Plasmid vector encoding his-tagged fluorescence protein was transformed in JM109(DE3) strain of Escherichia coli. Expression and purification by affinity chromatography were performed by using the TALON metal affinity resin (Takara Bio, CA, USA) according to the manufacturer's protocol. The purified fluorescence protein was concentrated to ~ 10 mg/ml by filtration and was stored at -80 ºC until use. For immobilization, fluorescence protein was prepared at 0.05 mg/ml in 20 mM HEPES (pH 9.0) containing 1 mM NaCl and 0.05% Triton-X surfactant. Triton-X surfactant is used to prevent nonspecific adsorption to the Su8 insulating layer and can be omitted when the background signal does not pose significant problems. Immobilization was performed by applying an electric field to the electrode in the presence of a protein solution. Most typically, square voltage pulses (amplitude: + 1.7 V; width: 100 ms, duty: 50%, number of pulses: 200) were applied versus the Ag/AgCl electrode placed in the bath using a source measurement unit (Keithley 2401, USA). The amount of protein immobilized is controllable through the voltage pulse condition (reference #17). The recording chamber was rinsed with pure water and filled with the recording solution. The chamber could be stored at 4 ºC for ~ 1 week, although the protein immobilization was normally done on the day of measurement. The following buffers adjusted with sodium hydroxide were used at 50 mM for pH titration: potassium hydrogen phthalate for pH 4 and 5, potassium dihydrogen phosphate for pH 6 and 7, and boric acid and potassium chloride for pH 8 and 9.

### Measurement and data analysis

Fluorescence imaging and electrochemical recording were performed in parallel by using the electrochemical analyzer (ALS 611E, CH Instruments, TX, USA) and the fluorescence microscope (Olympus BX51WI, Tokyo, Japan) equipped with a water immersion objective (60x), stable xenon lamp (Ushio, Tokyo, Japan), and the CMOS camera (C11440, Hamamatsu Photonics, Japan). The recording solution consisted of 20 mM HEPES (pH 7.4; adjusted with NaOH) and 1 mM NaCl. These instruments were synchronized with TTL pulses from a pulse generator (AWG50, Elmos, Osaka, Japan). The Ag/AgCl and platinum electrodes were used as the reference and working electrodes. Typically, the optical responses from fluorescence protein were reproducibly observed in the potential range between − 1.7 and + 1.5 V. At more negative potentials, the generation of vigorous hydrogen bulbs could damage the protein layer at the interface. The excitation and emission filters used were 497/16 nm and 535/25 nm (Semrock, NY, USA) for Venus fluorescence; 535/50 nm and 610/75 nm (Semrock, NY, USA) for dKeima. Images were acquired with HC-Image software (Hamamatsu Photonics, Japan), and analyzed using ImageJ (NIH, Japan) and the custom-made procedures of IDL (Research Systems, USA).

### Supplementary Information

Supplementary Information 1.

Supplementary Movie S1.

Supplementary Movie S2.

Supplementary Movie S3.

Supplementary Movie S4.

Supplementary Movie S5.

Supplementary Movie S6.

Supplementary Movie S7.

## Data Availability

The datasets generated during and/or analyzed during the current study are available from the corresponding author on reasonable request.
