# Supplementary material for: Reverse pH-dependent fluorescence protein visualizes pattern of interfacial proton dynamics during hydrogen evolution reaction
Source: Sci Rep. 2023 Oct 15;13:17489. doi: 10.1038/s41598-023-44758-4 (PMC10577132; doi:10.1038/s41598-023-44758-4)
Supplement: Supplementary file 1 — Supplementary Information 1. [file 41598_2023_44758_MOESM1_ESM.pdf]

Supplementary Information

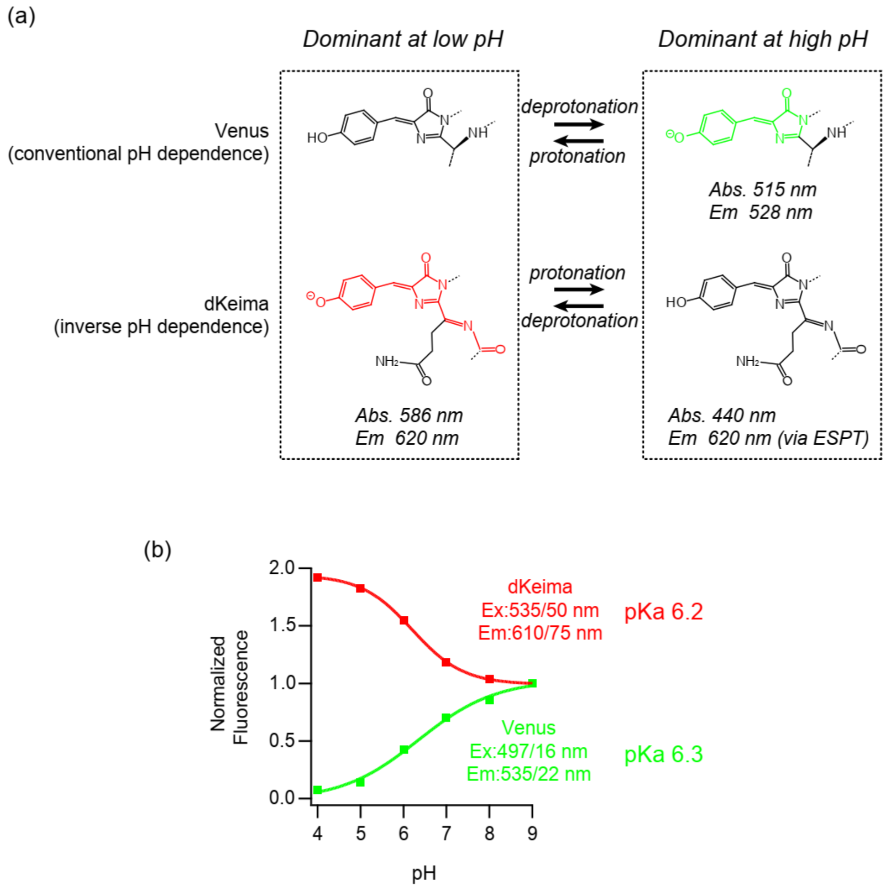

Figure S1. The chromophore protonation states in Venus and dKeima.
(a) A summary of the chromophore states in Venus and dKeima of the conventional and inverse pH dependence, respectively. ESPT: excited-state proton transfer. **(b)** Dependence on pH of Venus and dKeima immobilized on the gold substrate. The sigmoid function was used for curve fitting and pKa determination.

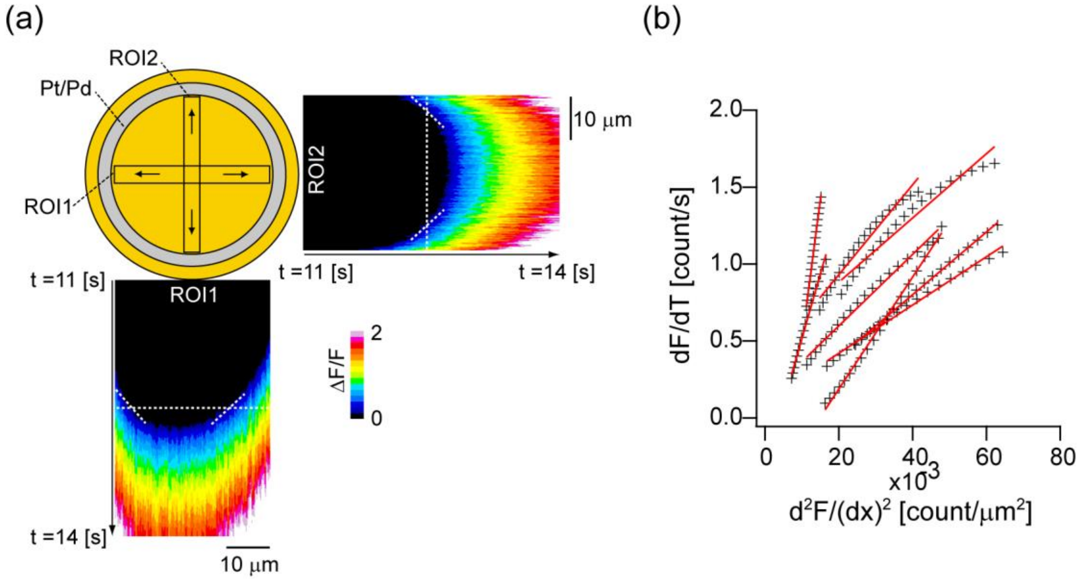

Figure S2. Image analysis of the propagating dKeima signal in the type #3 electrode. (**a**) To determine the propagation rate in the four directions (i.e. left, right, up, and down), the regions of interest (ROI1, 2) were set as indicated. The raster plots are the profiles of ROI 1 and 2 with a smooth filter applied and converted to normalized fluorescence change (ΔF/F) concerning data at t = 11 s. The propagation rates were determined from the slope for ΔF/F = 0.1 at t = 12.5 sec as indicated by the white dotted lines. (**b**) The slopes in the $d^2F/(dx)^2$ versus df/dt plot for the four directions at t = 12.2 and 12.3 sec were subject to the linear fits (8 slopes in total) to estimate the diffusion coefficient.

Supplementary Movies

Movie S1: Pseudo-colored presentation of dKeima signal (ΔF/F) in electrode #1.

Movie S2: Pseudo-colored presentation of dKeima signal (ΔF/F) in electrode #2.

Movie S3: Pseudo-colored presentation of dKeima signal (ΔF/F) in electrode #3.

Movie S4: Venus imaging in the split electrode (left = active; right = resting)

Movie S5: Venus imaging in the split electrode (left = resting; right = active)

Movie S6: dKeima imaging in the split electrode (left = active; right = resting)

Movie S7: dKeima imaging in the split electrode (left = resting; right = active)
